# Grain Boundary Structure Ordering via Electroshock Treatment Enhances Strength‐Ductility Synergy

**DOI:** 10.1002/advs.77128

**Published:** 2026-08-19

**Authors:** Jiancheng Chen, Feng Wang, Dongsheng Qian, Huiling Wang, Jinsong Wu, Mingwang Fu, Lin Hua

**Affiliations:** ^1^ State Key Laboratory of Light Superalloys Wuhan University of Technology Wuhan China; ^2^ Department of Mechanical Engineering Research Institute for Advanced Manufacturing The Hong Kong Polytechnic University Kowloon Hong Kong China; ^3^ School of Materials Science and Engineering Wuhan University of Technology Wuhan China; ^4^ Hubei Engineering Research Center for Green Precision Material Forming Wuhan China; ^5^ State Key Laboratory of Advanced Technology for Materials Synthesis and Processing Wuhan University of Technology Wuhan China; ^6^ Yangtze Laboratory Wuhan China

**Keywords:** dislocation, electroshock treatment, grain boundary, strength‐ductility synergy, structural transition

## Abstract

High‐energy electropulsing triggers recrystallization and phase transformation via excessive Joule heating, potentially causing unwanted damage and compromising the bulk microstructure. Herein, a non‐destructive, low‐energy electric current strategy, termed electroshock treatment (EST), is developed to strengthen grain boundaries (GBs), the preferential site for crack initiation and propagation, while preserving the bulk microstructure. Using α‐Ti as a model system, this strategy achieves a synergistic enhancement of strength and ductility by transforming the defect‐rich GB structure into an ordered atomic arrangement. In situ micropillar compression tests confirm a significant increase in GB strength after EST, while molecular dynamics (MD) simulations reveal that ordered GBs, owing to their lower density of defects, raise the critical stress for dislocation emission and exhibit greater resistance to intergranular fracture. Additionally, a reduction in dislocation density after EST contributes to enhanced ductility. These findings provide a promising GB‐structure‐engineering pathway to reinforce polycrystalline metallic materials.

## Graphical abstract

Electroshock treatment triggers atomic rearrangement, transforming disordered grain boundaries into ordered states without inducing recrystallization. This grain boundary ordering effectively relieves strain concentration and strengthens boundaries, enabling a simultaneous improvement in both strength and ductility.

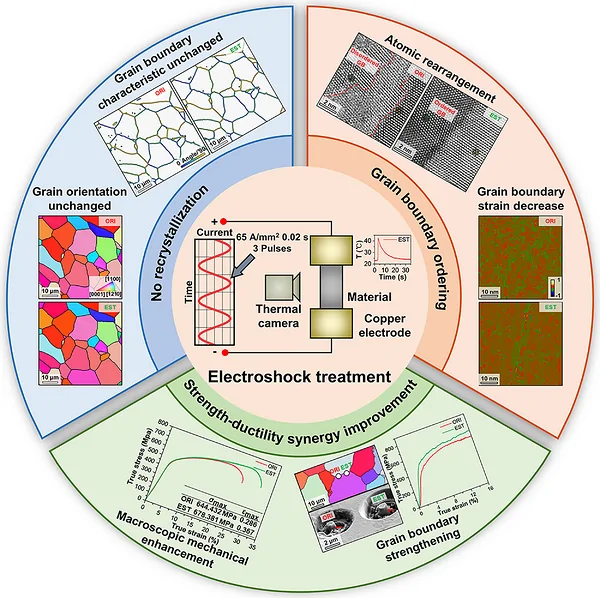

## 1 Introduction

High‐energy electropulsing treatment has emerged as an effective processing technique for strengthening bulk metallic materials [1, 2, 3]. These methods with high current densities or long action times trigger recrystallization and phase transformation through intense Joule heating [4, 5, 6, 7, 8, 9, 10]. By integrating electric current's athermal effects (e.g., electron wind force [11], electron‐dislocation interaction [12], and charge imbalance [13]) with rapid heating [14], recrystallization and phase transformation can be achieved at lower temperatures and faster rates than conventional heat treatment [15, 16, 17, 18, 19]. However, high‐energy electropulsing‐induced recrystallization and phase transformation will change the carefully engineered microstructures from prior manufacturing [20, 21]. The associated high temperatures may also introduce thermal damage that compromises the material's structural integrity. Consequently, there is a pressing need for a mild electric current strategy that enables lattice defect tailoring for performance improvement, while avoiding destructive microstructure evolution and thermal damage.

Grain boundaries (GBs), as the weakest links in polycrystalline materials, play a decisive role in determining mechanical properties [22, 23]. High‐energy electropulsing generates intense Joule heating to modify GB density and GB macroscopic characteristics (including rotation axis, rotation angle, and GB plane normal vector) in various advanced alloys, such as titanium alloys, nickel‐based superalloys, high‐entropy alloys, and steels [24, 25, 26, 27]. For instance, high‐energy electropulsing applied to nickel‐based superalloys not only refined the grain size but also induced the formation of high‐density twin boundaries [8]. Similarly, electropulsing treatment in steels was shown to increase GB density, resulting in the formation of more ultrafine/fine grains [28]. In addition to completely reconstructing GBs, GB structure tailoring also represents a valuable pathway to enhancing GB performance [29, 30, 31, 32].

GB structures can be broadly classified as disordered or ordered, with disordered GBs comprising two distinct categories: segregation‐induced equilibrium amorphous GBs [33, 34] and defect‐rich non‐equilibrium GBs [35, 36]. The segregation‐induced equilibrium amorphous interfaces, such as the nanoscale disordered intergranular layers formed by multielement cosegregation in superlattice alloys [33, 34], are thermodynamically stable and serve as ductile buffer layers that enhance dislocation accommodation and suppress intergranular fracture, thereby simultaneously improving strength and ductility. The defect‐rich non‐equilibrium GBs, where dislocation pile‐ups, point‐defect accumulation, and local stress concentration render the GB a preferential site for crack nucleation rather than a ductile buffer [35, 36]. This defect‐rich disordered state necessitates relaxation via ordering of the GB atomic arrangement (i.e., reducing local atomic disorder and GB energy at the interface). According to the trade‐off model for the strength‐ductility relationship [37], material failure is governed by interface cracking, where the interface strength determines the upper bound of the strength‐ductility combination. GB relaxation enhances interfacial cohesion, thereby enabling simultaneous improvements in strength and ductility. Under constant direct‐current annealing at ∼865 °C for 4 hours, electric current‐induced GB disorder‐to‐order structural transitions accompanied by abnormal grain growth have been observed in metal oxides [38, 39]. However, it remains unclear whether low‐energy electric current can stabilize GB atomic structures from a defect‐rich disordered state into an ordered one while preserving the GB macroscopic characteristics linked to the bulk microstructure in metals.

In this work, we propose a low‐power, short‐duration, temperature‐controlled electric current strategy, termed electroshock treatment (EST), which stabilizes GB structures while preserving the grain/phase microstructure, thus enabling a further enhancement of the strength–ductility synergy. Unlike high‐energy electropulsing methods that rely on large‐scale microstructural reconstruction, EST focuses on the tailoring of crystal defects. Using α‐Ti as a model system, we achieved the observation of GB atomic structures in macroscopic samples under EST, thereby directly revealing the correlation between them and macroscopic mechanical properties. In situ characterization revealed that EST induced a GB structure transition from disordered to ordered atomic arrangements. In situ GB mechanical testing and MD simulations have confirmed that the enhancement of GB strength and fracture resistance resulted from GB ordering. Ruling out the influence of dislocations under the EST, GB structure ordering contributes to the enhanced strength‐ductility synergy. These findings elucidate a novel GB strengthening mechanism driven by EST, offering a new GB‐structure‐engineering avenue to achieve superior strength‐ductility synergy in metals.

## 2 Results and Discussion

### 2.1 EST‐Induced Strength‐Ductility Synergy With Microstructural Stability

**FIGURE 1:**
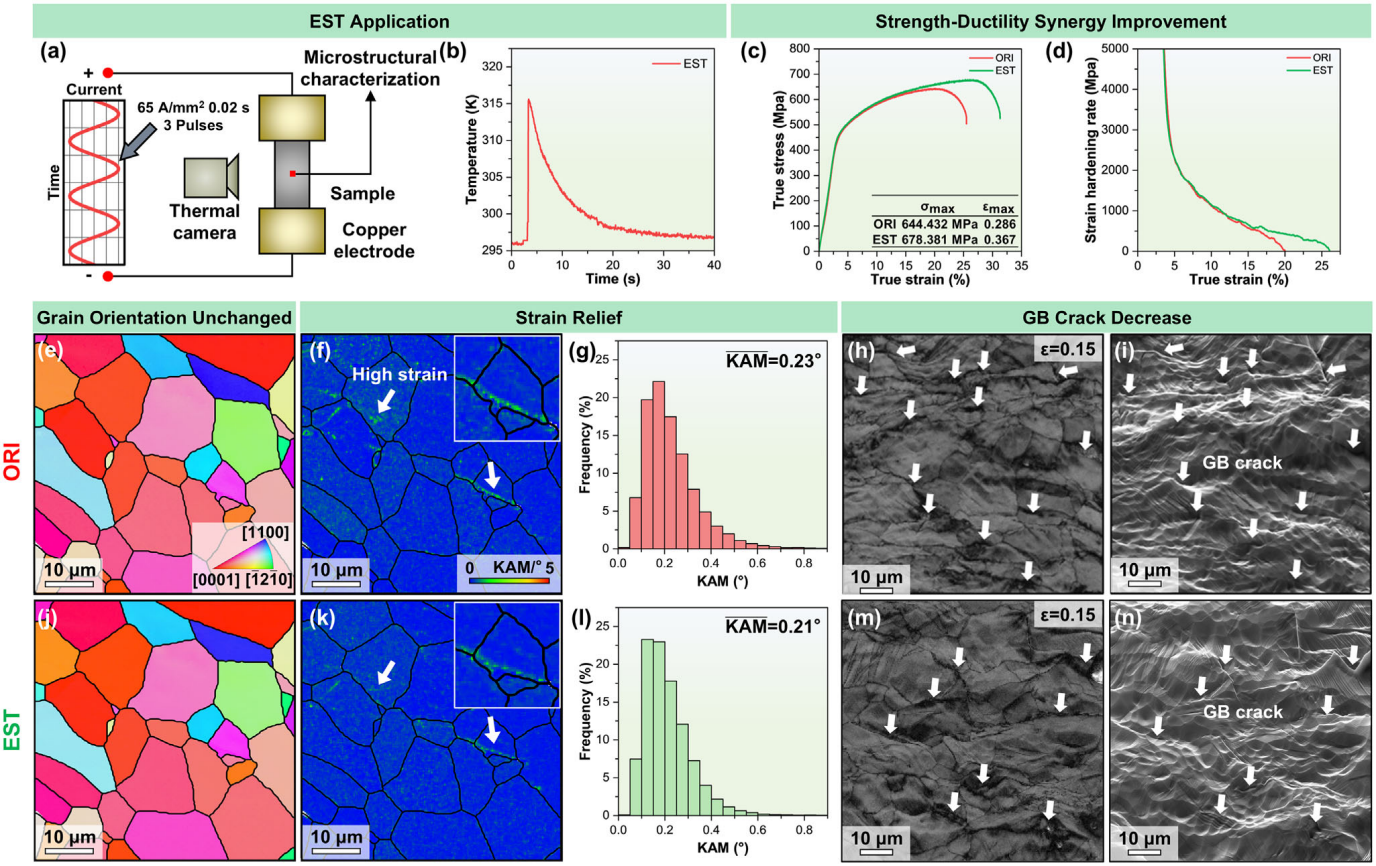
Evolution of mechanical performance and the corresponding microstructure changes in α‐Ti under EST. (a) Schematic illustration of the EST equipment. (b) Time‐temperature curve of the EST process. (c) Uniaxial tensile true stress‐strain curves for ORI and EST samples at room temperature. (d) Strain‐hardening rate versus true strain curves for ORI and EST samples. (e,j) In situ grain orientation in the z‐direction of the specimen coordinate system before and after EST. (f,k) In situ KAM analysis before and after EST. (g,l) KAM statistical chart corresponding to (f,k). (h,m) Band contrast maps at a strain of 0.15 illustrate GB distribution and the relatively darker regions representing high‐strain zones. (i,n) The SEM images corresponding to (h,m) reveal the location of GB cracks.

Bulk α‐Ti materials were subjected to the customized EST parameter shown in Figure 1a. The process is characterized by an ultra‐short duration (0.06 s) and a limited peak temperature (max. 316 K, Figure 1b), distinguishing it from high‐energy electropulsing (see Figure S1). The true stress–strain curves revealed a remarkable enhancement in mechanical performance: the ultimate tensile strength (UTS) increased from 644 MPa (Original, ORI) to 678 MPa (EST), while the uniform elongation (UE) improved significantly from 28.6% to 36.7% (Figure 1c). The work hardening curves (Figure 1d) further illustrate that, following the initial elastic‐plastic transition, the EST sample maintained a higher hardening rate for a longer strain duration, indicating a superior ability to accumulate plastic strain.

Crucially, this enhancement occurs without recrystallization and phase transformation. In situ inverse pole figure (IPF) maps (Figure 1e,j) confirmed that grain orientations and morphology remained unchanged after EST. This ruled out grain refinement, a common strengthening mechanism in high‐energy electropulsing [40, 41, 42], as the origin of the enhanced mechanical properties. Kernel average misorientation (KAM) maps revealed a selective reduction in lattice distortion. High‐distortion regions, particularly those concentrated near GBs, were significantly reduced after EST, while the low‐distortion grain interiors remained stable (Figure 1f,k). Quantitatively, the average KAM value decreased from 0.231 to 0.213 (Figure 1g,l), a trend corroborated by in situ X‐ray diffraction (XRD) showing a reduction in dislocation density from 1.06 × 10^15^ to 0.84 × 10^15^ m^−2^ (see Figure S2). Band contrast maps and corresponding scanning electron microscope (SEM) morphology (Figure 1h,i) further identified GB as the primary sites for stress concentration and microcrack initiation in the ORI sample. The suppression of GB cracking in the EST sample (Figure 1m,n) strongly implied that EST selectively heals high‐energy defects at the GBs, thereby enhancing their intrinsic strength. These observations identify GBs as the strength‐ductility bottleneck. Premature GB cracking terminates dislocation accumulation and work hardening before the material reaches its full capacity. However, due to limited spatial resolution, these microscale characterizations primarily reveal defect reduction in the GB region, necessitating further atomic‐scale observations to directly examine how changes in GB structure contribute to enhanced mechanical performance.

### 2.2 Disorder‐To‐Order Structural Transition of Grain Boundaries

**FIGURE 2:**
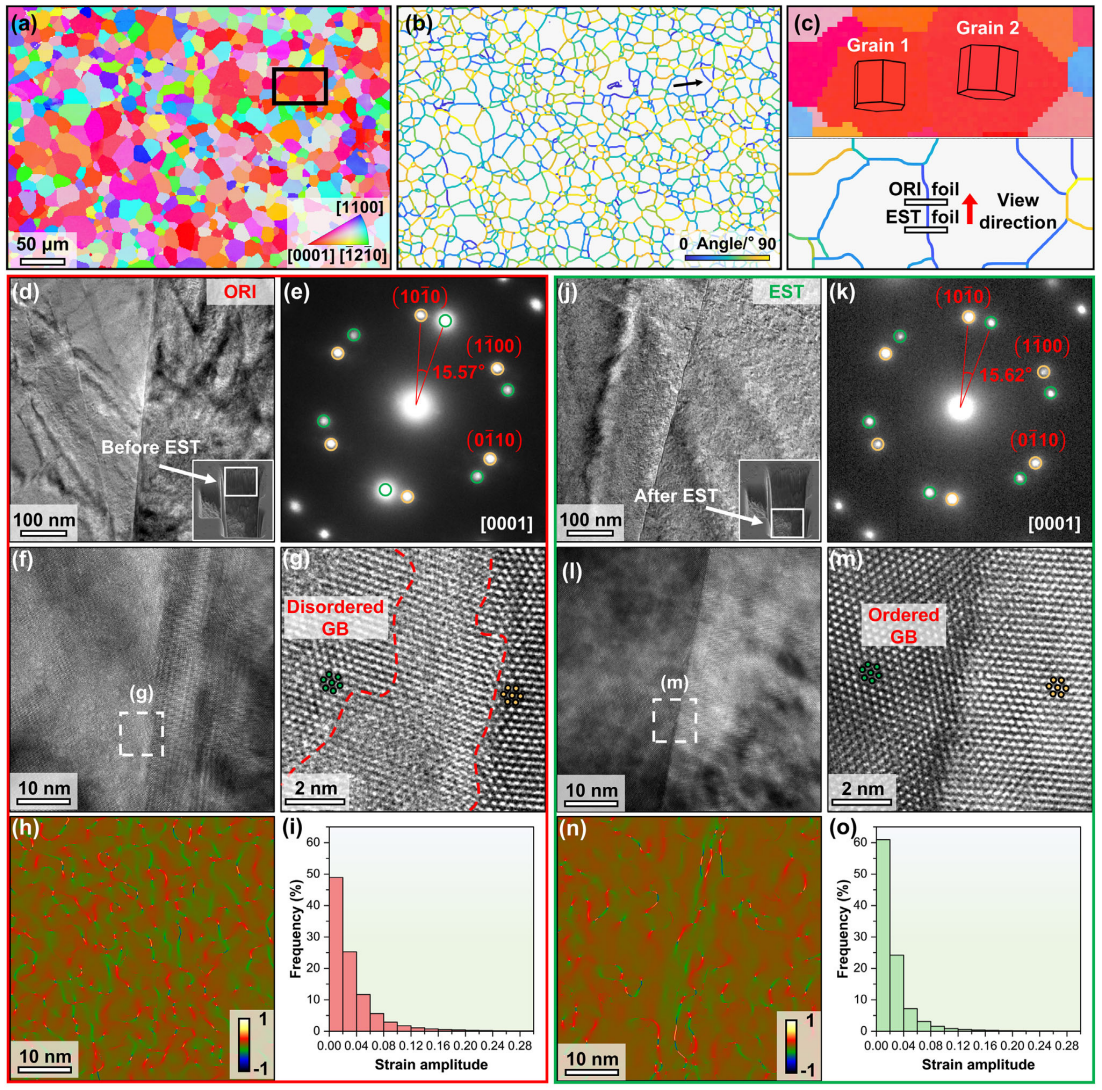
Comparison of the GB atomic structure between the ORI and EST states. (a) An IPF map along the Y direction. (b) GB angle map in the region shown in (a). (c) Magnified view of the IPF map at a near [0001] tilt GB located in the region shown in (a). In situ TEM observations were conducted on the same GB. The ORI foil was first prepared, and the EST foil was subsequently fabricated. The red arrow indicates the direction of TEM observation. (d,j) GB morphology in the ORI and EST samples, respectively. (e,k) Selected area electron diffraction (SAED) pattern along the [0001] zone axis in (d,j). The green‐ and yellow‐marked atomic arrangements correspond to the green‐ and yellow‐circled diffraction spots in the SAED pattern, respectively. (f,l) High‐resolution TEM (HRTEM) images of the GB region. White dot lines mark the local GB regions corresponding to (g,m). (g,m) HRTEM images of the local GB atomic structure for the ORI and EST samples. The red dashed lines indicate the boundaries of the structurally disordered GB region, determined by the transition from irregular atomic arrangements to periodic crystalline lattice fringes. (h,n) GPA mapping of the GB region corresponding to (f,l). The *ε*_xx_ strain component was mapped using the $(1\bar{1}00)\;$ and $\left(10\bar{1}0\right)$ diffraction spots. (i,o) GPA statistical chart corresponding to (h, n).

To elucidate the atomic‐scale origins of this strengthening, we performed a targeted analysis of GB structures. Bicrystals sharing a common [0001] zone axis were identified via electron backscattered diffraction (EBSD) to allow for in situ transmission electron microscopy (TEM) observation before and after EST (Figure 2a–c). Figure 2d,j presents two segments of the same GB. As shown in Figure 2e,k and Figure S3, the GB macroscopic characteristics (rotation axis and angle) remained invariant. However, at the atomic scale, a striking transformation was observed in Figure 2f,l (see also Figure S4). The GB structure transitioned from a disordered arrangement with high strain in the ORI state (Figure 2g,h) to an ordered, low‐strain configuration after EST (Figure 2m,n). Geometric phase analysis (GPA) performed on Figure 2f,l quantifies this “healing” effect: the proportion of low‐strain (<0.02) increased from 48.92% to 60.97% (Figure 2i,o). The phenomenon of GB atomic structure ordering without recrystallization and phase transformation was similarly observed in TC11 titanium alloy and 7075 aluminum alloy (see Figure S5), where grain morphology remained largely stable, dislocation density decreased significantly, and GB regions exhibited a more ordered arrangement.

The evolution mechanism behind this GB structural transition can be examined from both thermal and athermal perspectives. From the thermal perspective, although GBs exhibit higher electrical resistance than grain interiors, microstructure‐based finite element simulations have demonstrated that no localized temperature elevation occurs at GBs under electric current, owing to rapid heat conduction across the extremely short distance between the GB and the grain interior [13]. In the present case, the thermal diffusion length during a single EST pulse (430 µm, based on the Fourier heat conduction equation) far exceeds the grain size, and the absence of recrystallization after EST (Figure 1e,j) confirms that the local GB temperature remains far below the recrystallization temperature of Ti (550–650°C). From the athermal perspective, our previous density functional theory calculations have demonstrated that electric current exerts additional forces specifically on atoms in defect regions, while atoms in perfect lattice sites remain unaffected [43]. This selective enhancement of atomic mobility at defect sites drives the directional slip of lattice dislocations toward GBs, followed by the decomposition of extrinsic GB dislocations and the reorganization of GB dislocation networks. Therefore, GB evolution was achieved via the athermal effect of the electric current, thereby relaxing the high‐energy GB state without altering the grain morphology.

### 2.3 Enhanced Grain Boundary Strength

**FIGURE 3:**
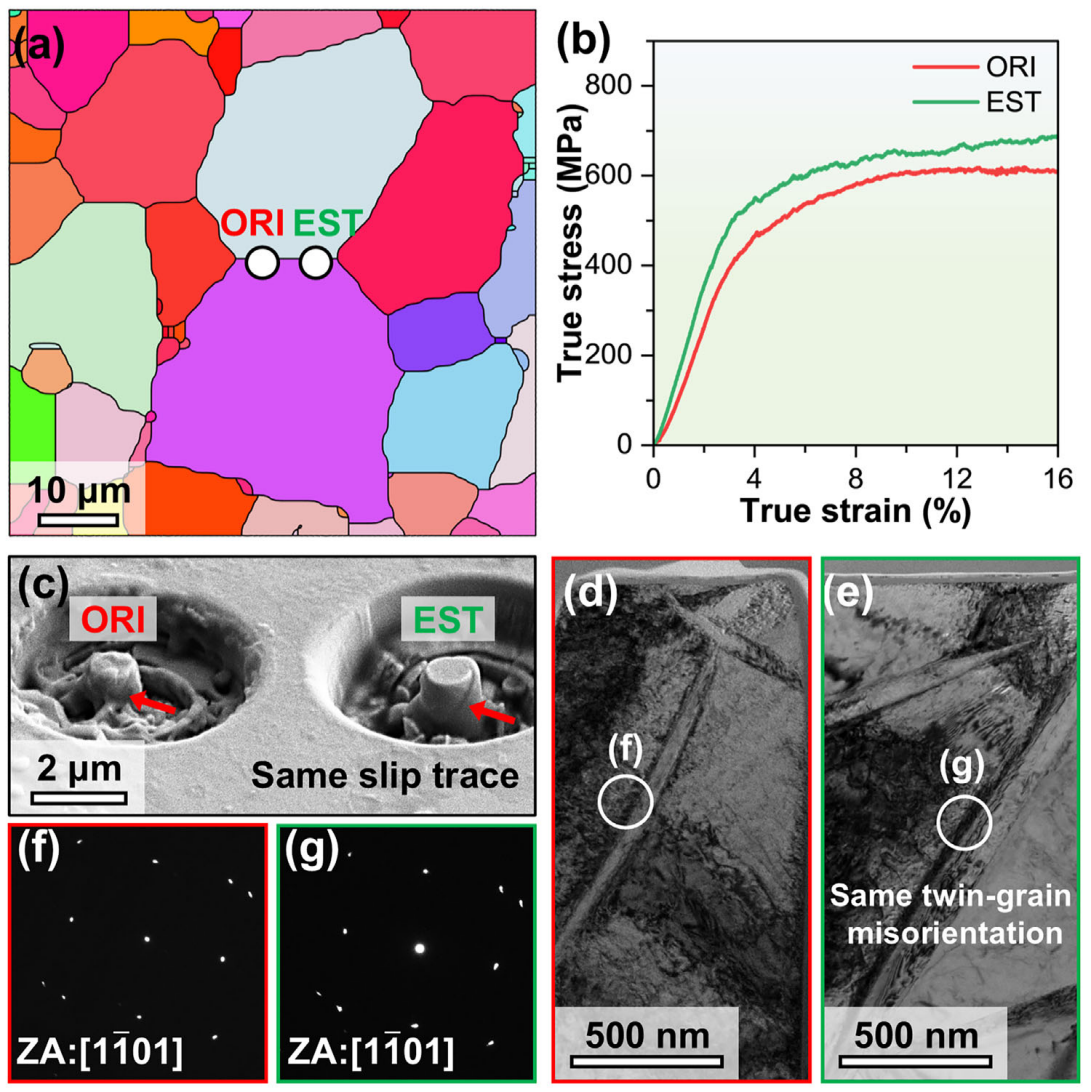
In situ micropillar compression tests conducted at a GB region. (a) IPF map along the Z direction used to select a GB for two micropillar compression tests, before and after EST, respectively. The white circles mark the sampling positions. (b) True stress–strain curves of the ORI and EST micropillars. (c) SEM image showing the morphology of deformed micropillars, with the red arrow indicating the slip trace. (d,e) TEM images illustrating the microstructures of the ORI and EST micropillars, respectively. SAED was performed at the regions marked by white circles. (f,g) SAED patterns taken at the grain–twin interface to show the orientation relationship. “ZA” is an abbreviation for “Zone Axis”.

To verify whether GB ordering relates to mechanical strengthening, we conducted in situ micropillar compression tests at the GB region (Figure 3a; Movie S1). To avoid artifacts caused by the unknown depth profile of the GB trace, micropillars were fabricated immediately adjacent to the GB to probe the mechanical response of the GB‐affected zone. Figure 3b shows the true stress‐strain curves of the ORI and EST micropillars. The EST micropillar exhibited a distinct increase in flow stress compared to the ORI micropillar (e.g., ∼688 vs. ∼611 MPa at >8% strain, Figure 3b). Figure 3c reveals that both micropillars exhibited the same slip traces after compression. Post‐compression TEM analysis (Figure 3d–g) confirmed that both samples deformed via similar slip and deformation twinning mechanisms, ruling out a change in deformation mode. Previous studies on α‐Ti micropillars suggest that pre‐existing lattice defects or stress concentrations lower the stress required for yield and twin nucleation [44, 45]. Consequently, the higher strength of the EST micropillar provides direct evidence that EST significantly reduced the content of structural defects (such as twin nuclei or dislocation tangles) in the GB region.

### 2.4 Atomistic Mechanism of GB Strengthening

**FIGURE 4:**
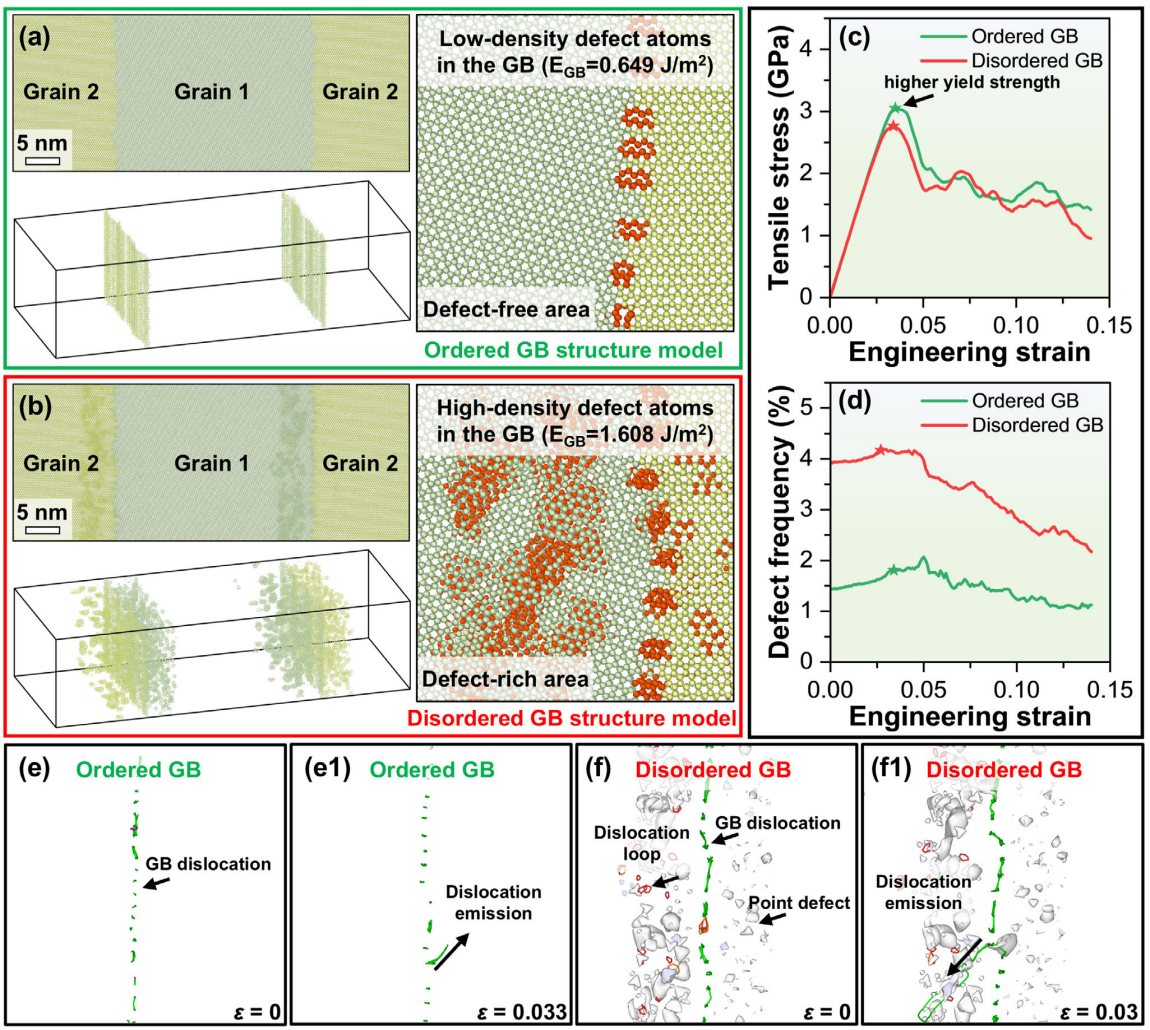
Tensile deformation simulation of ordered and disordered GB models. (a) Ordered GB model featuring low‐density defective atoms with a GB energy of E_GB_  =  0.649 J/m^2^. (b) Disordered GB model featuring high‐density defective atoms with E_GB_=  1.608 J/m^2^. (c) Engineering tensile stress–strain curves and (d) evolution of defect frequency as a function of strain for both models under a strain rate of 10^9^ s^−1^. The star symbols indicate the critical strains corresponding to the onset of dislocation emission. (e,e1) Snapshots of the ordered GB at strains of *ε* = 0 and *ε* = 0.033, capturing stress relief via dislocation emission. (f,f1) Snapshots of the disordered GB at strains of *ε* = 0 and *ε* = 0.03, highlighting earlier dislocation emission mediated by abundant point defects.

Molecular dynamics (MD) simulations were employed to compare the intrinsic strength and fracture resistance of ordered versus disordered GBs. Two distinct GB models, ordered and disordered, were constructed and distinguished by their GB energy and defective‐atom content. The ordered model comprises two low‐defect GBs with a lower grain boundary energy of E_GB_ =  0.649 J/m^2^ (Figure 4a), while the disordered model contains two disordered GBs characterized by a highly frustrated structural state and a higher energy of E_GB_ =  1.608 J/m^2^ (Figure 4b), both derived from bicrystals with the grain orientations specified in Figure 2. The disordered GB model was designed to represent the high‐density defect state characteristic of the ORI‐state GBs, while the ordered model corresponds to the relaxed configuration following EST. The uniaxial tensile deformation was simulated along the direction perpendicular to the GB plane under the NPT ensemble. As illustrated in the engineering tensile stress–strain curves (Figure 4c), a distinctly higher yield strength (∼3.0 GPa) is achieved in the ordered GB model compared to that of the disordered counterpart (∼2.7 GPa), followed by a gradual decrease in both models as extensive dislocation propagation effectively releases the accumulated strain energy and consumes interfacial defects. Furthermore, additional tensile simulations were performed under the NVT ensemble, which restricts lateral contraction and elevates the stress triaxiality (a condition analogous to the constrained stress state arising from strain incompatibility between differently oriented grains in polycrystalline materials). This elevated triaxiality promotes intergranular fracture within the accessible MD timescale, revealing that the ordered GB exhibits greater fracture resistance than the disordered GB (see Note S6).

Real‐time defect evolution at the GBs was tracked via a dislocation extraction algorithm [46] during tensile loading, revealing that both models accommodate plastic deformation through an identical primary mode: progressive defect accumulation at the GB with increasing stress until yielding, followed by stress relief via dislocation emission. The critical stress required to trigger dislocation emission, however, differs between the two models and is governed by the defect frequency (Figure 4d). The disordered GB, with a high initial defect frequency (∼4%, Figure 4f,f1), emits dislocations at a markedly lower stress and lower strain (*ε* = 0.03) than the ordered GB (initial defect frequency ∼1.5%; emission at *ε* = 0.033, Figure 4e,e1), leading to premature yielding.

### 2.5 Excluding Dislocation From the Strength‐Ductility Synergy Mechanism

**FIGURE 5:**
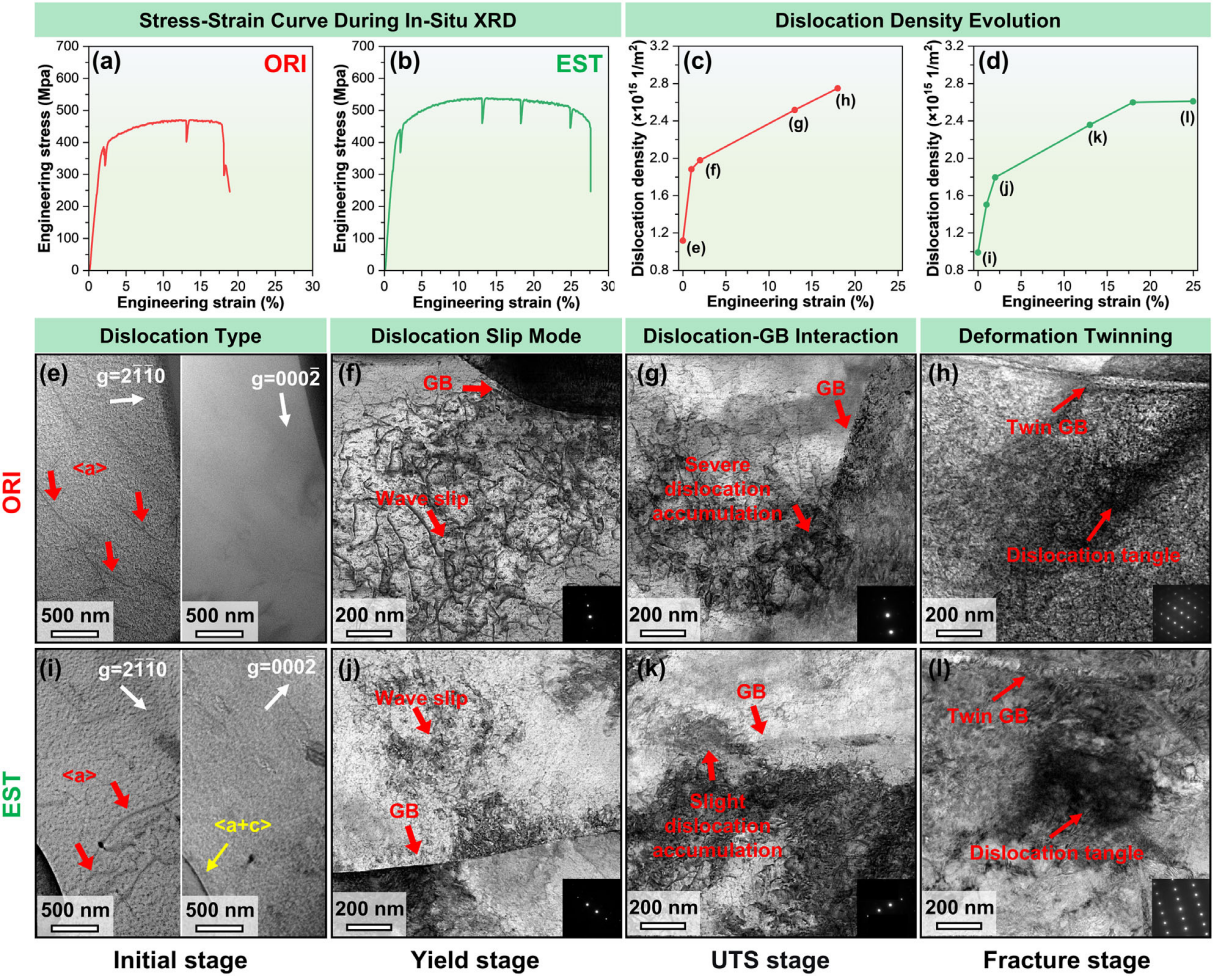
Comparison of dislocation evolutions between ORI and EST samples during tensile deformation. (a,b) In situ XRD tensile curve at room temperature and after EST. Each characteristic “serration” observed on the curves corresponds to an individual XRD measurement, while this feature was absent in the measurement during the elastic deformation stage. (c,d) Dislocation density versus engineering strain response derived from the in situ XRD patterns in Figure S7. (e–h) Dislocation configuration evolution of the ORI sample. (i–l) Dislocation configuration evolution of the EST sample. The dislocation configuration was captured at four strain stages: initial, yield, UTS, and fracture stages. Dislocation types of the undeformed ORI and EST samples were identified using diffraction vectors $g=2\bar{1}\bar{1}0$ and g  =  0002, respectively.

Finally, we evaluate the role of dislocation in the strength‐ductility synergy. The tensile curves (Figure 5a,b) from in situ XRD (see Figure S7) mirrored the macroscopic improvement. The dislocation density evolved similarly in both samples (Figure 5c,d), differing only in the EST sample's lower initial density (EST: 0.98 × 10^15^ m^−2^; ORI: 1.12 × 10^15^ m^−2^). Unlike the high‐energy electropulsing‐enhanced activation of <c + a> dislocations [47], the dominant dislocation type remained unchanged after EST (Figure 5e,i). Geometrically necessary dislocation analysis confirmed that <a>‐type dislocations dominated in both ORI and EST samples, and EST reduced the overall dislocation density without altering the relative proportions of dislocation types (see Figure S8). Both samples consistently exhibited a wavy slip mode at yield (Figure 5f,j), in contrast to the reported slip‐mode alteration by electropulsing‐assisted deformation [48]. At the UTS stage, the lower dislocation accumulation near GBs in the EST sample (Figure 5g,k) indicated that its GBs more effectively alleviate stress concentration through active dislocation‐mediated mechanisms [49, 50, 51, 52]. By the fracture stage, the consistent dislocation‐twin interaction (Figure 5h,j) was not responsible for the variation in performance [53]. Furthermore, in situ tensile EBSD (see Figure S9) confirmed that the sequence of dislocation evolution was identical in both samples, with the EST sample exhibiting consistently lower KAM values throughout the deformation process.

The contribution of dislocations to strength‐ductility synergy depends on intrinsic slip resistance and the critical spacing of activated slip planes [37]. Intrinsic slip resistance is mainly improved by alloying‐induced compositional fluctuations [54, 55], but EST has no effect on it, as pure Ti has negligible alloying elements and EST adds no new ones. A smaller critical spacing between activated slip planes corresponds to more slip bands inside grains and more uniform shear deformation, which is linked to dislocation slip modes [48, 56, 57, 58]. However, EST reduced the initial dislocation density, whereas other characteristics were unaffected, including the proportion of dislocation types and the evolution of dislocation density, distribution, and morphology. In addition, the initial reduction in dislocation density induced by EST resulted in a trade‐off between strength loss and ductility improvement [59]. Therefore, while the dislocation density reduction contributed to ductility, it did not constitute a strength‐ductility synergy mechanism. The observed synergy is attributed to GB structure ordering, which enhanced interfacial strength and enabled simultaneous improvements in both strength and ductility. This ordering is directly confirmed by in situ HRTEM. Furthermore, in situ GB micropillar compression reveals a ∼12.6% increase in flow stress after EST. MD simulations provide atomistic insight suggesting that the ordered GBs, owing to their lower defect density, raise the critical stress for dislocation emission and exhibit greater resistance to intergranular fracture. The strengthened GBs suppress GB cracking, thereby delaying failure and sustaining work hardening.

## 3 Conclusions

In summary, we demonstrate that EST enhances strength‐ductility synergy in metallic materials by tailoring GB structure. Unlike high‐energy electropulsing, which is unsuitable for application to the earlier‐optimized bulk microstructure, EST preserves the original grain orientation and morphology. Mechanistically, EST induces a GB disorder‐to‐order structural transition and effectively releases concentrated lattice strain. In situ mechanical testing confirms the enhanced GB strength after EST, while MD simulations reveal that ordered GBs have fewer defects, thereby increasing the critical stress required for dislocation emission and enhancing resistance to intergranular fracture. The reduction in dislocation density induced by EST, with the types and motion behavior of dislocations remaining unchanged, results in a strength‐ductility trade‐off rather than synergy. Verified by characterization on various polycrystalline metals, including pure titanium, TC11 titanium alloy, and 7075 aluminum alloy, this work establishes EST as a potent, non‐destructive post‐processing strategy for reinforcing polycrystalline metallic materials via GB structure ordering.

## 4 Materials and Methods

### 4.1 Materials

In this work, commercial pure Ti (Grade 2) in the annealed state (typical annealing at 650–700°C for 1–2 h, followed by air cooling) was used, with its chemical composition provided in Table S2. The Ti bars were cut into rectangular samples with dimensions of 5 × 10 × 15 mm^3^. Then, the samples were treated by EST. The self‐made EST generator is shown in Figure 1a. The experimental current density was 65 A/mm^2^; the number of pulses was set to 3, and the pulse frequency was 50 Hz. The infrared camera (Fotric 226) was used to measure the maximum temperature rise of the samples under EST, and the temperature rise for EST parameters is shown in Figure 1b. TC11 titanium alloy and 7075 aluminum alloy were used for additional microstructure characterization.

### 4.2 Tensile Testing

The as‐received and EST samples were tested to evaluate mechanical properties. Uniaxial tensile tests were conducted on a universal testing machine (SUNS WDW‐50) using dogbone‐shaped specimens with a gauge length of 12 mm. The tests were performed at a nominal strain rate of 3 × 10^−3^ s^−1^ at ambient temperature. Five specimens were tested for each condition to ensure reproducibility. The dogbone‐shaped tensile samples were prepared using electrical discharge machining, and the damaged surface layer was removed via mechanical polishing prior to mechanical testing.

### 4.3 Microstructure Analysis

In situ EBSD (Oxford Nordly Max2) was employed to evaluate the microstructural changes induced by EST. The specimens were electrochemically polished in a solution consisting of 5 mL perchloric acid and 45 mL glacial acetic acid. The microstructures of the samples were characterized by EBSD on the 10 × 15 mm^2^ face, and the EBSD data were processed using MTEX software [60]. EBSD mapping was performed at an acceleration voltage of 20 kV with a step size of 0.3 µm. Following characterization of the original state, the sample was subjected to EST treatment, after which EBSD characterization was conducted again.

To evaluate the dislocation density variation induced by EST, in situ XRD measurements were performed on the 10×15 mm^2^ face using an X‐ray diffractometer (Empyrean). Following characterization of the original state, the sample was subjected to EST treatment, after which XRD characterization was repeated. The 2θ scan range was set to 30°–90°. The modified Williamson–Hall (mWH) and modified Warren‐Averbach (mWA) methods were adopted to evaluate the dislocation densities of the titanium samples (see Note S2).

The deformation behaviors of the ORI and EST samples with varying degrees of deformation were observed by FEI Talos F200X TEM. TEM samples were prepared using an FEI Helios NanoLab G3 UC Focused Ion Beam (FIB) system under standard conditions. The TEM foils were cut and thinned using a Ga^+^ ion beam operated at 30 kV, with a final ion current of 80 pA applied for taper removal and surface polishing. Dislocation structures were determined under two‐beam conditions with different diffraction vectors $\vec{g}$. Based on the tensile test results, as‐received and EST samples with varying degrees of deformation (including the original state, yield point, maximum tensile point, near‐fracture state, and fracture state) were prepared to analyze the tensile deformation mechanism.

The in situ tensile testing was combined with EBSD (Oxford Symmetry) to examine dislocation configurations and with XRD (MicroMax‐007HF) to evaluate dislocation densities. During the EBSD measurements, scans were taken at strain levels of 0%, 2%, 5%, 10%, and 15%. The test was performed at ambient temperature and a stretching speed of 0.033 mm/min. For the XRD analysis, five critical deformation stages were selected: the original stage, elastic stage, yield point, peak tensile stress, and near‐fracture stage. These in situ XRD tensile tests were carried out at room temperature under a strain rate of 6 × 10^−4^ s^−1^, using a Mo target operated at 50 kV and 24 mA.

To characterize the GB structure, the EBSD‐FIB technique was employed to ensure that the two sides of the GB share a similar crystal zone axis. This allows for the observation of atomic structures on both sides of the GB via TEM. The first step was to conduct multiple‐area EBSD mappings, with a step size of 0.3 µm, over a total area of 1 mm^2^. Then, an appropriate GB was sought according to the IPF, where the adjacent grains had closely aligned [0001] orientations, and this direction was taken as the zone axis for TEM observation. An IPF‐Y map showing the crystallographic orientations of the α phase is presented in Figure 2a. Figure 2b displays the misorientation angles for all GBs. The selected GB was a [0001] tilt GB, where the angle between the [0001] vector of the two grains was only 3.37°—small enough to support HRTEM observation along this zone axis (Figure 2c). Additionally, its length was sufficient to allow cutting into two different segments. The misorientation angle of this GB is approximately 15.6°. The in situ experimental procedure was as follows: Vickers hardness indentations were first introduced on the sample surface as markers to define the EBSD scanning regions. The target GB was then located by correlating the EBSD map with the corresponding SEM surface morphology, and a FIB cut was made. After EST treatment, the same GB was relocated using the identical procedure, and a second FIB cut was performed at an adjacent position along the same GB. The consistency of the SAED patterns before and after EST (Figure 2e,k) confirmed that both foils were extracted from the same GB with identical misorientation characteristics. A Digital Micrograph plugin was used to perform GPA for microscopic strain analysis [61].

### 4.4 In Situ Micropillar Compression Testing

The influence of EST on the GB mechanical performance was investigated using in situ micropillar compression tests at room temperature, performed on an FT‐NMT04 nanoindentation system integrated into a Crossbeam 550 SEM. Initially, a sufficiently long GB was selected based on EBSD analysis. Subsequently, two adjacent micropillars with diameters of ∼1 µm and heights of ∼2 µm were fabricated at the GB location, one before and the other after EST. This aspect ratio was selected to prevent buckling while ensuring a uniform stress state along the micropillar height [62, 63]. Compression tests were conducted at a constant strain rate of 1×10^−3^ s^−^^1^ until the maximum strain reached ∼16%. To characterize the morphology of micropillars, foils were prepared from the compressed micropillars by sectioning perpendicular to the GB plane for TEM observation.

### 4.5 MD Simulation

MD simulations were performed on Ti bicrystal models containing two GBs at the center, with grain orientations determined from the bicrystal observed in TEM (grain 1: (0, 0, −14.6°); grain 2: (0, 0, 1°)). Periodic boundary conditions were applied in all three directions. For each rotated grain, rectangular supercell matrices were enumerated using a Diophantine‐based approach, and the transverse dimensions were matched between the two grains within a tolerance of 0.1 Å to ensure lattice compatibility across the periodic boundaries. Two GB models with distinct structural states were constructed. The ordered GB model comprised two low‐defect GBs with a GB energy of E_GB_ = 0.649 J/m^2^. The disordered GB model was constructed by removing atoms from one side of the GB and adding an equal number of atoms to the other side, followed by energy minimization and structural relaxation, yielding a highly frustrated structural state with E_GB_ = 1.608 J/m^2^. The interaction between Ti atoms was represented using the EAM potential developed by Mendelev et al. [64]. Prior to loading, all models were optimized via conjugate gradient minimization and then equilibrated at 300 K for 500 ps under an isothermal‐isobaric ensemble (NPT) with a Nosé‐Hoover thermostat/barostat and a timestep of 1 fs. For the primary tensile simulations, double GB models (∼590 × 230 × 150 Å^3^, ∼1 200 000 atoms) were used. Uniaxial tension was applied along the direction perpendicular to the GB plane under an NPT ensemble with the transverse pressures maintained at zero, at a constant strain rate of 10^9^ s^−1^. Although the strain rate used in MD simulations (10^9^ s^−1^) is orders of magnitude higher than experimental values (∼10^−3^ s^−1^), both GB models were compared under identical conditions, ensuring that the relative trends in defect evolution are qualitatively valid. To evaluate GB fracture behavior, additional simulations were performed under the thermostat (NVT) ensemble with identical strain rate and loading parameters, which restricts lateral contraction and produces a stress state with elevated stress triaxiality. Structural defects during deformation were identified and quantified using the polyhedral template matching method [65], and the defect frequency was defined as the ratio of defective atoms to the total number of atoms. All MD calculations were conducted with the LAMMPS code [66], and visualization was carried out using OVITO software [67].

## Author Contributions

**Jiancheng Chen**: methodology, investigation, writing – original draft, data curation, writing – review and editing, visualization. **Feng Wang**: conceptualization, supervision, writing – review and editing, formal analysis. **Dongsheng Qian**: data curation, investigation, funding acquisition. **Huiling Wang**: formal analysis, writing – original draft, investigation, validation, visualization. **Jinsong Wu**: writing – review and editing, supervision. **Lin Hua**: conceptualization, supervision, funding acquisition, project administration. **Mingwang Fu**: writing – review and editing, supervision.

## Conflicts of Interest

The authors declare no conflicts of interest.

## Supporting information




**Supporting File 1**: advs77128‐sup‐0001‐SuppMat.docx.

**Supporting File 2**: advs77128‐sup‐0002‐Movie1.mp4.

## Data Availability

The data that support the findings of this study are available from the corresponding author upon reasonable request.
