# Supplementary material for: Grain Boundary Structure Ordering via Electroshock Treatment Enhances Strength‐Ductility Synergy
Source: Adv Sci (Weinh). 2026 Aug 19:e77128. Online ahead of print. doi: 10.1002/advs.77128 (PMC13487870; doi:10.1002/advs.77128)
Supplement: Supplementary file 1 [file ADVS-9999-e77128-s001.docx]

# Supplementary Materials for “Grain boundary structure ordering via electroshock treatment enhances strength-ductility synergy”

Jiancheng Chen^1,2,3,4,#^, Feng Wang^1,3,4,#^, Dongsheng Qian^1,3,4,*^, Huiling Wang^1,3,4^, Jinsong Wu^5,6,*^, Mingwang Fu^2,*^ , Lin Hua^1,3,4,*^

^1^ State Key Laboratory of Light Superalloys, Wuhan University of Technology, Wuhan, 430070, China

^2^ Department of Mechanical Engineering, Research Institute for Advanced Manufacturing, The Hong Kong Polytechnic University, Hung Hom, Kowloon, Hong Kong, China

^3^ School of Materials Science and Engineering, Wuhan University of Technology, Wuhan, 430070, China

^4^ Hubei Engineering Research Center for Green Precision Material Forming, Wuhan, 430070, China

^5^ State Key Laboratory of Advanced Technology for Materials Synthesis and Processing, Wuhan University of Technology, Wuhan, 430070, China.

^6^ Yangtze Laboratory, Wuhan, 430205, China

^#^ These authors contributed equally: Jiancheng Chen, Feng Wang.

^*^Corresponding authors: qiands@whut.edu.cn; wujs@whut.edu.cn; mmmwfu@polyu.edu.hk; hualin@whut.edu.cn

## Contents:

Supplementary Figure 1. Partial parameters of various electric current strategies.

Supplementary Figure 2. Dislocation density calculation.

Supplementary Figure 3. The GB macroscopic degree of freedom corresponding to Figure 2(e, j).

Supplementary Figure 4. GB Structure before and after EST.

Supplementary Figure 5. Characterization data on other metallic materials processed by EST.

Supplementary Figure 6. Tensile deformation simulation of ordered and disordered GB models under NVT ensemble.

Supplementary Figure 7. In-situ XRD patterns of the ORI and EST sample at different engineering strains along the loading direction during deformation.

Supplementary Figure 8. Comparison of the dislocation type evolution of the ORI and EST samples at 0% and 15% strain.

Supplementary Figure 9. Comparison of the dislocation distribution evolution of the ORI and EST samples.

Supplementary Table 1. Partial parameters of various electric current strategies.

Supplementary Table 2. Chemical composition of CP Ti.

Supplementary Note 1. Partial parameters of various electric current strategies

Supplementary Note 2. Dislocation density calculation

Supplementary Note 3. GB macroscopic degree of freedom

Supplementary Note 4. GB structure before and after EST

Supplementary Note 5. Characterization data on other metallic materials processed by EST

Supplementary Note 6. Grain boundary fracture resistance under elevated stress triaxiality (NVT)

Supplementary Note 7. In-situ XRD patterns

Supplementary Note 8. Dislocation configuration evolution analysis via in-situ tensile EBSD

## Supplementary Note 1. Partial parameters of various electric current strategies

**Supplementary Figure 1. Partial parameters of various electric current strategies ^1-24^.**

**Supplementary Table 1.** **Partial parameters of various electric current strategies.**

| **Material** | **J (A/mm^2^)** | **t (s)** | **T (K)** | **Recrystallization/**  **Phase transformation** |
| --- | --- | --- | --- | --- |
| **Pure Ti (This work)** | **65** | **0.06** | **316** | **No** |
| TC11^[1]^ | 100 | 0.225 | 353 | No |
| Aluminum clad steel^[2]^ | 150 | 0.5 | 623 | No |
| Pure Ti^[3]^ | 7220 | 0.0008 | 903 | Yes |
| Pure Ti^[4]^ | 22 | 4 | 1023 | Yes |
| Pure Ti^[5]^ | 29.1 | 4 | 1243 | Yes |
| Pure Ti^[6]^ | 88 | 0.4 | 1346 | Yes |
| TC4^[7]^ | 27.6 | 3.72 | 1175 | Yes |
| TC4^[8]^ | 65.5 | 0.34 | 1173 | Yes |
| TC4^[9]^ | 65.5 | 0.4 | 1290 | Yes |
| TC4^[10]^ | 83.4 | 0.44 | 1253 | Yes |
| TC4^[11]^ | 140 | 0.075 | 1341 | Yes |
| TC4^[12]^ | 56 | 0.46 | 1346 | Yes |
| TC4^[13]^ | 133 | 28.1 | 1282 | Yes |
| Fe-18Mn-10.5Al-1C-6Ni^[14]^ | 7500 | 0.00031 | 619 | Yes |
| Al_0.1_CoCrFeNi^[15]^ | 90 | 5 | 863 | Yes |
| Al-Cu-Li alloy^[16]^ | 60 | 10 | 713 | Yes |
| Cu-3.5Ti alloy^[17]^ | 20.3 | 10 | 923 | Yes |
| Mg-Gd-Y-Zn-Zr alloy^[18]^ | 30 | 300 | 543 | Yes |
| 1045 steel^[19]^ | 55 | 600 | 573 | Yes |
| Mg-RE-Zn alloy^[20]^ | 805 | 600 | 683 | Yes |
| AZ31 alloy^[21]^ | 1880 | 30 | 716 | Yes |
| 718Plus alloy^[22]^ | 83 | 600 | 1373 | Yes |
| GH3030 alloy^[23]^ | 9.2 | 1200 | 1123 | Yes |
| nickel-based superalloy^[24]^ | 13.7 | 1800 | 1273 | Yes |

## Supplementary Note 2. Dislocation density calculation

In this work, the modified Williamson-Hall and modified Warren-Averbach (mWH/mWA) methods were adopted to evaluate the dislocation densities of alpha-Ti^[25]^. The modified Williamson-Hall (mWH) method is given as:

$\Delta K=0.9/D+\left( \frac{\pi M^{2}b^{2}\rho}{2} \right)^{\frac{1}{2}}\left( K\bar{C}^{\frac{1}{2}} \right)+O\left( K^{2}\bar{C} \right)$

where *D* is the volume averaged crystal size, *M* is a parameter associated with the effective outer cut-off radius of dislocations, *b* is the magnitude of the Burgers vector, *ρ* is the dislocation density, and *K* is the Bragg position, $\bar{C}$ is the average contrast factor of dislocations, $\Delta K = \beta_{hk.l} cos \theta/\lambda$ and $K = 2 sin \theta/\lambda$. β_hk.l_ is the full width at half maximum (FWHM) of peak broadening, $\theta$ is the diffraction angle and *λ* is the wavelength of X-rays. Generally, the contrast factor of dislocations for each diffraction peak can be estimated by $C_{hk.0}$, $q_{1}$ and $q_{2}$ values. For hexagonal crystals, the contrast factor of dislocations is given as:

$C_{hk.l}=C_{hk.0}\left( 1+q_{1}x+q_{2}x^{2} \right)$

$x=(2/3)(l/ga)^{2}$

where $C_{hk.0}$ is the average contrast factor for {hk.0} reflections, $q_{1}$ and $q_{2}$ are parameters related to the anisotropic elastic constants of metals. $l$ is the fourth index of the {hk.l} reflection, g is the magnitude of the diffraction vector, and a is the lattice parameter. Substituting Eq. (2) into (1), the following equation can be obtained:

$(\Delta K-\alpha)^{2}/K^{2}=\beta(1+q_{1}x+q_{2}x^{2})$

where α = 0.9 /D and $\beta=\frac{\pi M^{2}b^{2}\rho}{2}\bar{C_{hk.0}b^{2}}$. By performing the best quadratic fit between $(\Delta K-\alpha)^{2}/K^{2}$ and $x$ via Eq. (4), $\alpha$, $\beta$, $q_{1}$, and $q_{2}$ can be obtained. Then, the fractions of the three Burgers vector types (i.e. < a > , < c >, and < c + a > ) of alpha-Ti can be calculated through the following equations^[26]^ :

$q_{1}=\left( \sum_{i=1}^{3} h_{i}\bar{C}_{hk.0}^{\left( i \right)}b_{i}^{2}q_{1}^{\left( i \right)} \right)/\left( \sum_{i=1}^{3} h_{i}\bar{C}_{hk.0}^{\left( i \right)}b_{i}^{2} \right)$

$q_{2}=\left( \sum_{i=1}^{3} h_{i}\bar{C}_{hk.0}^{\left( i \right)}b_{i}^{2}q_{2}^{\left( i \right)} \right)/\left( \sum_{i=1}^{3} h_{i}\bar{C}_{hk.0}^{\left( i \right)}b_{i}^{2} \right)$

$\sum_{i=1}^{3} h_{i}=1$

In addition, h_i_ should satisfy the basic condition: 0 < h_i_< 1. Where h_i_ and b_i_ are the fractions and Burgers vector magnitude of <a>, <c> and <c + a> dislocations, respectively. The theoretical values of $\bar{C}_{hk.0}^{\left( i \right)}$, $q_{1}^{\left( i \right)}$and $q_{2}^{\left( i \right)}$of Ti are from the reference^[27]^. After the fractions of h_i_ are obtained, the value of $\bar{C_{hk.l}b^{2}}$ can be calculated and the mWA method can be applied^[28]^ :

$\bar{C_{hk.l}b^{2}}=\sum_{i=1}^{3} h_{i}\bar{C}_{hk.0}\left( 1+q_{1}x+q_{2}x^{2} \right)b_{i}^{2}$

$\ln A\left( L \right)=\ln A^{s}\left( L \right)-Y\left( L \right)\left( K^{2}\bar{C_{hk.l}b^{2}} \right)+O\left( K^{4}\bar{C_{hk.l}^{2}b^{4}} \right)$

$Y\left( L \right)=-\left( \rho\pi L^{2}/2 \right)\ln\left( R_{e}/L \right)$

where L, $A\left( L \right)$ and $A^{s}\left( L \right)$stand for the Fourier parameter, the Fourier coefficients of the diffraction profile, and the size Fourier coefficients, respectively. Then, Eq. (10) can be transformed into:

$Y\left( L \right)/L^{2}=\left( \pi\rho/2 \right)\ln L-\left( \pi\rho/2 \right)\ln R_{e}$

Finally, the dislocation density of alpha-Ti can be calculated by determining the slope between Y (L) / L^2^ and ln L. The peak profiles before and after EST are presented in **Supplementary Figure 2(a)**. The dislocation density was determined by fitting Eq. (11) to the plot of $\ln L$ versus $Y\left( L \right)/L^{2}$, as shown in **Supplementary Figure 2(b)**. A comparison reveals that the dislocation density decreased from 1.06×10^15^ m^-2^ in the initial state to 0.84×10^15^ m^-2^ after EST.

**Supplementary Figure 2.** **Dislocation density calculation.** (a) In-situ XRD patterns of the sample before and after EST. (b) modified Warren-Averbach plots obtained from line profile in the XRD diffraction patterns of the sample before and after EST.

## Supplementary Note 3. GB macroscopic degree of freedom

**Supplementary Figure 3****. The GB macroscopic degree of freedom corresponding to Figure 2(e, j).** (a, c) GB axes map of the sample before and after EST. (b, d) GB angle map of the sample before and after EST.

## Supplementary Note 4. GB structure before and after EST

**Supplementary Figure 4.** **GB Structure of ORI and EST states.** (a, e) GB morphology in the ORI and EST samples, respectively. (b-d) Disordered GB in the ORI state sample. (f-h) Ordered GB in the EST state sample.

## Supplementary Note 5. Characterization data on other metallic materials processed by EST

**Supplementary Figure 5. Characterization data on other metallic materials processed by EST.** The EST of TC11 titanium alloy (a, b) and 7075 aluminum alloy (c, d) shows consistent microstructural evolution behavior: grain morphology remains largely stable, dislocation density decreases significantly, and GB regions display a more ordered arrangement.

## Supplementary Note 6. GB fracture resistance under elevated stress triaxiality (NVT)

To probe GB fracture behavior, additional tensile simulations were performed under the NVT ensemble, which restricts lateral contraction and elevates the stress triaxiality at the GB, a condition analogous to the constrained stress state at GBs in polycrystalline materials. Two configurations were employed. The first contained two ordered GBs and served as the low-defect baseline (**Supplementary Figure 6(a)**). The second deliberately combined one ordered and one disordered GB (**Supplementary Figure 6(b)**), so that the competition between the two GB types could be resolved directly within one simulation under identical loading. Both configurations adopted the grain orientations specified in **Figure 2**.

**Supplementary Figure 6(a, b)** showed that fracture occurred intergranularly in both configurations. The tensile curves (**Supplementary Figure 6(c)**) revealed that the ordered GB model fractured at *ε* = 0.13, whereas the disordered GB model failed prematurely at *ε* = 0.125. Since both models shared identical grain orientations and crystal structures, the flow stress during plastic deformation was governed by the same dislocation nucleation and propagation processes within the grain interiors, resulting in similar stress–strain responses prior to fracture. Energy and defect evolution analysis (**Supplementary Figure 6 (d, e))** revealed that both ordered and disordered GBs underwent self-relaxation via dislocation emission. However, the disordered GB, with a higher initial defect density, accumulated defects more rapidly during deformation and reached the critical fracture threshold earlier, whereas the ordered GB maintained a lower defect content and energy before failure. These results demonstrate that the EST-induced ordered GB exhibits greater resistance to intergranular fracture.

**Supplementary Figure 6. Tensile deformation simulation of ordered and disordered GB models under NVT ensemble.** (a) An ordered GB model with two ordered GBs, exhibiting intergranular cracks at both GBs when *ε* = 0.13. (b) A disordered GB model containing both ordered and disordered GBs, with cracking occurring along the disordered GB at *ε* = 0.125. (c) Stress–strain curves of both ordered and disordered GB models under a strain rate of 10^9^ s^−1^. (d) GB energy versus strain response for the ordered and disordered GBs in the disordered GB model. (e) The evolution of defect number at the GBs with strain for both ordered and disordered GBs in the disordered GB model.

## Supplementary Note 7. In-situ XRD patterns

**Supplementary Figure 7. In-situ XRD patterns of the ORI and EST sample at different engineering strains along the loading direction during deformation.**

## Supplementary Note 8. Dislocation configuration evolution analysis via in-situ tensile EBSD

To further investigate deformation mechanisms of ORI and EST samples during the early-to-mid stages of plastic deformation (corresponding to the curve range preceding 15% true strain in **Figure 1(c)**, an in-situ tensile EBSD technique was used to characterize the dislocation distribution evolution. The distribution of geometrically necessary dislocation (GND) densities in both ORI and EST samples at 0% strain is shown in **Supplementary Figure 8**. It can be observed that in **Supplementary Figure 8(a)**, the dislocations are predominantly of the <a> type (with an average density of 7.28×10^13^ m^-2^). Some <c+a>-type dislocations are also present (average density of 2.46×10^13^ m^-2^), while <c>-type dislocations are nearly absent. This observation indicates that the GND distribution is consistent with the dislocation configuration observed in **Figure 5** via TEM. In the EST sample, the density of <a>-type dislocations reached 5.74×10^13^ m^-2^, and the density of <c+a>-type dislocations reached 2.33×10^13^ m^-2^, both of which are lower than those in the ORI sample.

**Supplementary Figure 9(a, b)** presents the initial grain orientation distribution of the ORI and EST samples in IPF maps. **Supplementary Figure 9(a1, b1)** shows KAM maps of the ORI and EST samples at different strains. At the initial deformation stage (*ε* = 0), strain localization was observed in both samples, manifested as linear green-colored features distributed primarily within the grain interiors. As strain increased to *ε* = 0.05, plastic deformation became significantly concentrated at the GBs, as exemplified by the grains highlighted in the red dot box. As the tensile deformation continues, the plastic deformation within the grains increases. At a strain of *ε* = 0.15, high KAM values had spread to encompass nearly the entire observed region, concurrent with a significant reduction in EBSD indexing resolution. Crucially, the sequence of dislocation evolution during this early-to-mid stage showed minimal difference between ORI and EST samples. Both exhibited identical behavior: dislocations initially accumulated and piled up at GBs, then increased in grain interiors, eventually pervading the grains. Concurrently, analysis of the average KAM evolution revealed similar trends in strain accumulation rate for both samples. However, the KAM values for the EST sample were consistently slightly lower than those for the ORI sample. Consistent with the in-situ observations presented in **Figure 1(g, l)**, this quantitative difference can be attributed to the EST.

**Supplementary Figure 8. Comparison of the dislocation type** **evolution of the ORI and EST samples at 0% and 15% strain.** (a) <a>, <c>, <c+a> type GND density distribution of the ORI sample. (b) <a>, <c>, <c+a> type dislocation density distribution of the EST sample.

**Supplementary Figure 9.** **Comparison of dislocation distribution evolution of the ORI and EST samples.** (a, b) IPFs of microstructures of the ORI and EST samples. (a1, b1) KAM maps of microstructures of the ORI and EST samples at different strains.

**Supplementary Table 2. Chemical composition of CP Ti.**

| Elements | Ti | Fe | C | N | O | H |
| --- | --- | --- | --- | --- | --- | --- |
| Content(wt.%) | Bal. | 0.3 | 0.1 | 0.03 | 0.25 | 0.015 |

## Supplementary references

[1]	Ren X, Z Wang, R An. A promising approach to enhance fatigue life of TC11 titanium alloy: Low dislocation density and surface grain refinement induced by electropulsing. *Journal of Materials Science & Technology* **204**, 60–70 (2025).

[2]	Zhang S, M Geng, M Kim, J-H Bae, H Nam Han, *et al.* Prolonged fatigue life in aluminum clad steel by electropulsing treatment: Retardation of interface-microcrack formation. *International Journal of Fatigue* **167**, 107376 (2023).

[3]	Song H, Z Wang. Improvement of mechanical properties of cold-rolled commercially pure Ti sheet by high density electropulsing. *Transactions of Nonferrous Metals Society of China* **22**, 1350–1355 (2012).

[4]	Lee SH, MH Bae, J Yu, S Cheon, JW Won, *et al.* Electropulsing anisotropy of cold-rolled Grade 2 titanium sheet: Effect of electric current direction on recrystallization and hardness. *Journal of Materials Research and Technology* **31**, 2249–2256 (2024).

[5]	Lee M, J Yu, MH Bae, JW Won, T Lee. Accelerated recrystallization behavior of commercially pure titanium subjected to an alternating-current electropulse. *Journal of Materials Research and Technology* **15**, 5706–5711 (2021).

[6]	Zhang S, H Choi, J Wang, Z Liu, HN Han, *et al.* Rapid crack healing and fatigue life extension in single-edge notched pure titanium via electropulsing treatment. *International Journal of Fatigue* **198**, 108967 (2025).

[7]	Zhou Y, X Xu, Y Zhao, X Yan, L Wei, *et al.* Introducing ω and O′ nanodomains in Ti-6Al-4V: The mechanism of accelerating α → β transformation kinetics via electropulsing. *Journal of Materials Science & Technology* **162**, 109–117 (2023).

[8]	Yan X, X Xu, Y Zhou, Z Wu, L Wei, *et al.* Constructing a novel bi-lamellar microstructure in selective laser melted Ti-6Al-4V alloy via electropulsing for improvement of strength and corrosion resistance. *Journal of Materials Science & Technology* **193**, 37–50 (2024).

[9]	Xu X, L Wei, X Yan, Y Yang, Y Yu, *et al.* Electropulsing-assisted chemical boundary engineering to achieve strengthening and toughening of Ti-6Al-4V alloy manufactured via laser powder bed fusion. *Materials Science and Engineering: A* **929**, 148130 (2025).

[10]	Zhou Y, X Xu, Y Zhao, X Yan, C Wu, *et al.* A novel strategy for preparation of α′ + β Dual-phase microstructure through heating massive-like transformation induced by Electropulsing in Ti-6Al-4V Alloy. *Scripta Materialia* **224**, 115156 (2023).

[11]	Zhang J, J Wang, Y Yan, F Wang, J Wang, *et al.* Boosting Strength and ductility of Ti-6Al-4V alloy via millisecond-level electric treatment induced heterogeneous nanostructures. *Acta Materialia* **307**, 121976 (2026).

[12]	Xu X, X Yan, Y Qian, X Chong, Y Zhou, *et al.* Ti-6Al-4V alloy strengthening via instantaneous phase transformation induced by electropulsing. *Journal of Alloys and Compounds* **899**, 163303 (2022).

[13]	Lu H, L Zhang, W Deng, J Cai, K Luo, *et al.* Influence of electropulsing treatment on microstructural evolution and tribological behaviour of laser directed energy deposited Ti-6Al-4V alloy. *Tribology International* **214**, 111333 (2026).

[14]	Kumar RP, NCS Srinivas, RK Pandey, R Manna. Electropulsing of low-density duplex steel for strengthening and ductilization by microstructural refinement. *Journal of Materials Processing Technology* **329**, 118423 (2024).

[15]	Wang Y, W Wu, F Cui, X Yang. Electropulsing treatment-induced the enhanced recrystallization of deformed Al0.1CoCrFeNi high-entropy alloy at lower temperature. *Journal of Materials Research and Technology* **25**, 912–924 (2023).

[16]	Li M, Y Zu, G Chen, Y Li, X Li. Ultrafast recrystallization and grain refinement of 2A97 Al-Cu-Li alloy by electropulsing assisted annealing at lower temperature. *Journal of Alloys and Compounds* **1003**, 175662 (2024).

[17]	Wang L, X Meng, Y Jiang, M Wang, J Zhang, *et al.* Electropulsing rapidly refined the microstructure and enhanced the strength and plasticity of Cu-3.5Ti alloy. *Journal of Alloys and Compounds* **1005**, 175974 (2024).

[18]	Zhang M, Y Ma, Y Zheng, J Zou, X Gao, *et al.* Enhancement mechanism of strength-plasticity synergy induced by electropulsing treatment in as-extruded Mg-Gd-Y-Zn-Zr wire. *Materials Characterization* **228**, 115437 (2025).

[19]	Li Z, Y Jiang, P La, J Miao, X Qiang, *et al.* Superior mechanical properties of heterostructure 1045 steel obtained by cold rolling and subsequent electropulsing treatment. *Materials & Design* **251**, 113651 (2025).

[20]	Ma D, C Xu, S Sui, C Wu, Z Zhang, *et al.* Accelerated phase transformation in wire arc additive manufactured Mg-RE-Zn alloys via electropulsing treatment. *Materials Characterization* **227**, 115312 (2025).

[21]	Shan Z, J Bai, J Fan, H Wu, H Zhang, *et al.* Exceptional mechanical properties of AZ31 alloy wire by combination of cold drawing and EPT. *Journal of Materials Science & Technology* **51**, 111–118 (2020).

[22]	Zhang Z, J Liu, X Luo, J Yan, J Guo, *et al.* Electropulsing-induced high-density annealing twins: A novel strategy to enhance ductility in additively manufactured 718Plus alloy. *Journal of Materials Science & Technology* **253**, 268–279 (2026).

[23]	Zhang H, C Zhang, B Han, J Qiu, H Li, *et al.* Evolution of grain boundary character distributions in a cold-deformed Nickel-based superalloy during electropulsing treatment. *Journal of Materials Research and Technology* **9**, 5723–5734 (2020).

[24]	Hao J, S Qin, L Yan, X Zhang. Breaking thermodynamic and kinetic barriers in superalloy homogenization process by electropulsing to improve mechanical properties. *Journal of Alloys and Compounds* **873**, 159854 (2021).

[25]	Ungár T, A Borbély. The effect of dislocation contrast on x-ray line broadening: A new approach to line profile analysis. *Applied Physics Letters* **69**, 3173–3175 (1996).

[26]	Máthis K, K Nyilas, A Axt, I Dragomir-Cernatescu, T Ungár, *et al.* The evolution of non-basal dislocations as a function of deformation temperature in pure magnesium determined by X-ray diffraction. *Acta Materialia* **52**, 2889–2894 (2004).

[27]	Dragomir IC, T Ungár. Contrast factors of dislocations in the hexagonal crystal system. *Journal of Applied Crystallography* **35**, 556–564 (2002).

[28]	Ungár T, S Ott, PG Sanders, A Borbély, JR Weertman. Dislocations, grain size and planar faults in nanostructured copper determined by high resolution X-ray diffraction and a new procedure of peak profile analysis. *Acta Materialia* **46**, 3693–3699 (1998).
